# Chagasic Esophagopathy With Achalasia and Megaesophagus: A Case Report

**DOI:** 10.1155/crdi/9930462

**Published:** 2026-04-22

**Authors:** Gabriel Xerez de Oliveira, Beatriz Lacerda Bezerra, José Antonio da Silva Júnior, Ellany Gurgel Cosme do Nascimento, Cléber de Mesquita Andrade

**Affiliations:** ^1^ Biomedical Sciences Department, Universidade do Estado do Rio Grande do Norte, Mossoró, Rio Grande do Norte, Brazil, uern.br

**Keywords:** achalasia, case reports, Chagas disease, dysphagia, megaesophagus

## Abstract

Chagasic esophagopathy, resulting from Trypanosoma cruzi‐induced myenteric plexus denervation, leads to lower esophageal sphincter hypertonia, esophageal body hypertrophy, and dilation (megaesophagus), manifesting as progressive dysphagia and regurgitation that impair nutritional status and quality of life. This case report aims to describe chronic esophageal dysphagia in a patient from an endemic area in northeastern Brazil, highlighting diagnostic and therapeutic challenges within the public health system, including delays due to COVID‐19 restrictions and resource limitations. A 67‐year‐old female from Rio Grande do Norte, with prior Chagas disease diagnosis (2004, etiologically treated), hypertension, and dyslipidemia, presented in 2019 with solid‐food dysphagia and nocturnal regurgitation. Initial tests (chest X‐ray, ECG, and echocardiography) were normal, but contrast studies (barium esophagogram, and colon) were delayed until 2022, revealing 7 cm esophageal dilation (Rezende Group III), cardia narrowing, and retained contrast/food residues. Symptoms worsened, with a 5 kg weight loss and liquid‐only diet. Videolaparoscopic Heller–Pinotti cardiomyotomy was performed in September 2023 after clinical optimization (isosorbide dinitrate and domperidone). The 2 h procedure was uneventful; discharge occurred within 24 h on zero diet, progressing to normal intake by 5 weeks. By February 2024, she was asymptomatic, with a 2 kg weight gain (from 66 kg). This case confirms laparoscopic cardiomyotomy’s efficacy (90% success in Rezende I–III) for symptom resolution in nonadvanced chagasic megaesophagus. Public health insights underscore Chagas underreporting, late diagnosis, and public health system barriers in vulnerable populations, advocating early serological screening and etiological treatment in acute phases to prevent chronic digestive forms in endemic regions.

## 1. Introduction

Chagas disease, caused by Trypanosoma cruzi, remains a neglected tropical disease with persistent challenges in early diagnosis of its digestive form, particularly in endemic regions with limited resources such as northeastern Brazil [1]. Despite advances in serological testing, underreporting and suboptimal epidemiological surveillance result in late detection [2]. In resource‐constrained public health systems like Brazil’s SUS, barriers including exam unavailability, financial constraints, and disruptions like the COVID‐19 pandemic exacerbate delays, allowing progression from asymptomatic chronic infection to advanced digestive manifestations.

Once infected, the patient may develop the acute phase of the disease, later progressing to the chronic phase. Chagasic esophagopathy exemplifies these issues: myenteric plexus denervation leads to lower esophageal sphincter hypertonia, esophageal dilation (megaesophagus), and progressive dysphagia, regurgitation, weight loss, and malnutrition—consequences of delayed evaluation that severely impair quality of life and increase morbidity [3], only an estimate of 8.1% of the patients develop the digestive form [4]. Late diagnosis not only misses opportunities for preventive etiological therapy but also advances disease to stages requiring invasive interventions, where surgical outcomes are still favorable but quality‐adjusted life years are compromised.

In the reported case, the primary aim is to discuss a patient from an endemic region with a probably late serological diagnosis that further developed chronic dysphagia, being diagnosed with chagasic esophagopathy, followed up until after surgical treatment.

## 2. Case Report

This is a qualitative and retrospective study, conducted with patient’s authorization and signed consent form, approved by the State University of Rio Grande do Norte Research Ethics Committee under approval no. 6.527.976/2023. The patient’s data were collected mainly through direct medical appointments and clinical records. A literature review was made to correlate clinical aspects of the disease with the patient’s case.

A 67‐year‐old female from Rio Grande do Norte, Northeast Brazil, attended for an initial checkup in the Chagas Disease Outpatient Clinic in the Medical School of State University of Rio Grande do Norte in 2019. She reported dysphagia when eating solid food associated with regurgitation episodes that used to occur mainly in the evening after dinner. Reported normal intestinal habits and denied any cardiovascular symptoms. As main pathological history, she had a previous diagnosis of Chagas Disease in 2004, hypertension, and dyslipidemia in regular treatment with antihypertensives and statin. The patient’s family history had both parents diagnosed with Chagas’ disease, and the two of them passed away due to cardiac conditions. Given the family history, a patient underwent serological tests for Chagas disease and is currently asymptomatic, with no report of acute symptoms. The physical examination did not have any relevant claims.

**FIGURE 1:**
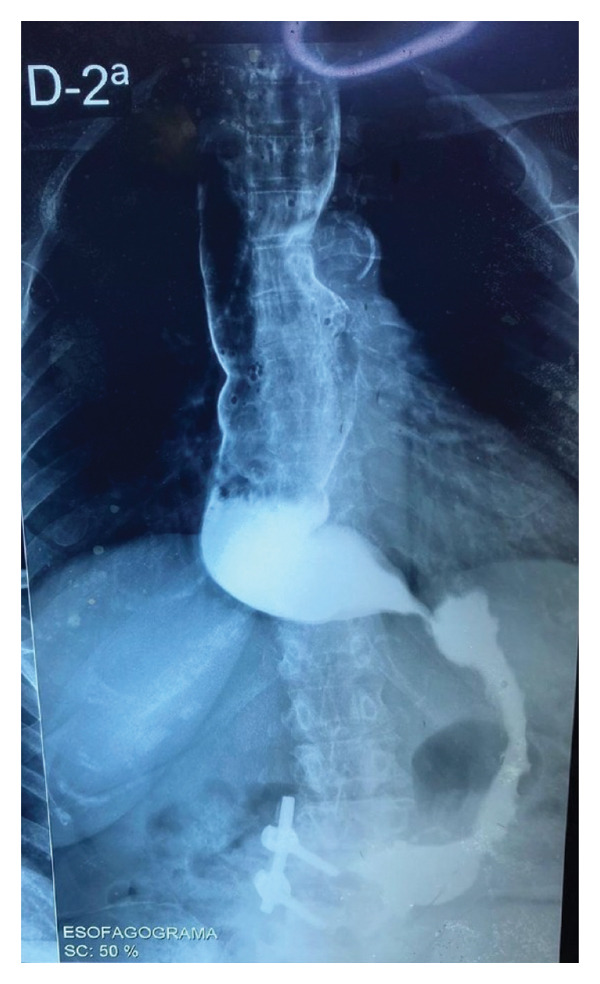
Esophagogram 1 min after contrast swallow. Posteroanterior view. (Source: author’s collection).

**FIGURE 2:**
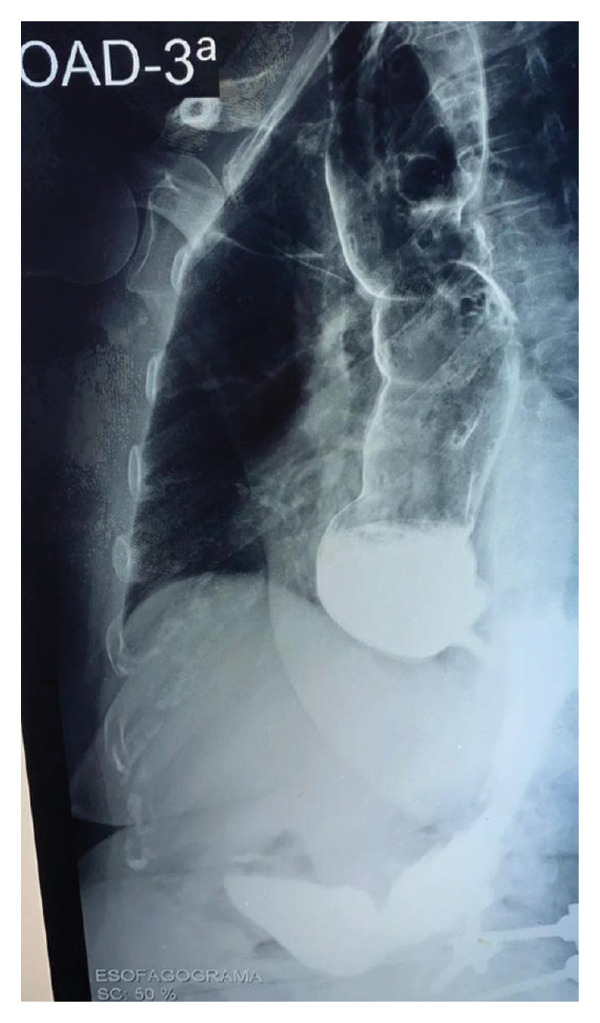
Esophagogram 1 minute after contrast swallow. Right anterior oblique (RAO) view. (Source: author’s collection).

The initial approach consisted of requesting chest radiography, electrocardiography, echocardiography, esophagogram with barium, and contrast‐enhanced colon radiography. Electrocardiography and echocardiography revealed no abnormalities; however, due to the unavailability of these examinations within the public health system, financial constraints, and restrictions imposed during the COVID‐19 pandemic, the patient was only able to return for the contrast‐enhanced studies three years later, in 2022. The esophagogram showed a dilation of 7 cm of the esophageal body, with caliber reduction in the cardia region associated with important retention of contrast, gastric fluids, and food content (Figures 1 and 2). At that moment she also reported worsening of the symptoms and nutritional status, with a total incapacity to eat solid food.

In January 2023, the patient sought care at a digestive surgery service at the School Hospital of the Federal University of Rio Grande do Norte, where surgical treatment was recommended. In the prescription, isosorbide dinitrate 5 mg and domperidone 10 mg were added, both to be taken minutes before meals to improve esophageal emptying. According to the patient’s reports, the medications were tolerable and helped in reducing symptoms of dysphagia and regurgitation.

In September 2023, four years after the first examination, the patient underwent Heller–Pinotti cardiomyotomy surgery at a private facility. The videolaparoscopic procedure was uneventful and lasted approximately 2 hours. Before surgery, she weighed 66 kg. At the postoperative period, she was admitted to the ward, remaining on a zero‐diet regimen until discharge within 24 h. After discharge, the diet was gradually reintroduced, and 5 weeks later she returned to her regular diet and was able to eat normally, without dysphagia, when consuming liquids or solids. In February 2024, she remained free of esophageal complaints or any other digestive symptoms, showing a weight gain of 2 kg compared to her weight before surgery. Table 1 provides a summary of the timeline of the case studied in this article.

**TABLE 1:** Summarizing the timeline of the case.

Event	Interventions/exams	Symptoms/outcome
2004		
Diagnosis and etiological treatment of Chagas’ disease	Serological tests	None
2019		
First consultation	Chest X‐ray, ECG, echocardiogram (normal); esophagogram and colonography requested	Onset of solid food dysphagia and nocturnal regurgitation
2022		
Esophagogram performed	Esophagogram and colonography performed	Dilation 7 cm (Rezende Group III), contrast retention/residue, 5 kg weight loss, liquid diet only
2023 Jan		
Referral for digestive surgery	Start with isosorbide dinitrate 5 mg + domperidone 10 mg before meals	Partial improvement of symptoms
Sep 2023		
Heller–Pinotti laparoscopic surgery		Starting with a zero‐calorie diet, gradual progression
Feb 2024		
Return to normal diet		No dysphagia for solids/liquids

## 3. Discussion

In Chagasic esophagopathy, the primary and most prominent symptom is the slowly progressive dysphagia, which may take years to develop. Besides, liquid intake becomes required to enable food motion through the esophagus [5], as reported by the patient. Complaints of heartburn are also common and suggest an inflammatory process in the esophageal mucosa, related to poorly digested food gathered in the distal esophagus [5].

Additionally, a mild involuntary weight loss was noticed over the period of 1 year, amounting to a total loss of 5 kg. This finding is consistent with the literature, which shows that approximately 60% of patients, when diagnosed with achalasia, experience some degree of weight loss due to ineffective esophageal emptying and reduced food intake [6].

Progressive dysphagia limited her eating capacity to the extent that significant complaints regarding compromised quality of life were reported during the appointments. Although an objective measurement tool was not applied, the patient’s subjective perception illustrates another aspect of the disease, in which the patients affected reported poor quality of life and health perception [7].

The patient clinical portrait could be explained by the denervation of inhibitory fibers in the myenteric plexus of Auerbach. This leads to a predominance of excitatory activity on smooth muscle cells of the esophagus [3]. The result is hypertonia and loss of receptive relaxation of the lower esophageal sphincter. Therefore, to overcome this increased pressure, the upstream esophageal body becomes hypertrophic and dilated, resulting in a remodeled organ known as megaesophagus [8]. All Chagas’s patients can suffer this denervation, even when asymptomatic, but to develop a typical megaesophagus, a 90% impairment of nerve fibers is generally necessary [3].

Between the onset of symptoms and the surgical treatment for megaesophagus, which had already progressed to Rezende’s Group III (Rezende’s classification III: 7–10 cm of esophagus dilatation at the barium esophagogram exam), approximately four years passed. It is important to state that the degree of dilation observed in the patient does not necessarily reflect a prolonged disease and does not correlate with the severity of symptoms [9, 10]. The long interval before treatment cannot be inferred as a causal factor for the progression of the megaesophagus or worsening of dysphagia. Furthermore, there are cases where patients with significant dilations experience only mild dysphagia, while others suffer with severe swallowing disorders and are classified in initial Rezende’s groups [10].

Another aspect to discuss is that the patient’s achalasia symptoms took approximately 15 years to appear, but there is no temporal pattern for their onset. There are cases of esophagopathy with rapid manifestation or even fast progress to advanced Rezende’s groups [6], while others remain stable in initial stages, with documented case reports that symptoms took decades to clinically appear [11].

Regarding the etiological treatment performed for the patient around 2004, there is evidence that timely diagnosis of Chagas disease, combined with adequate etiological treatment, can reduce or prevent the progression to the cardiac form [12]. This did not happen in the patient’s case, because despite etiological treatment, she progressed to the digestive form of the disease. A late serological diagnosis could explain this outline by leading to a delayed etiological treatment when the esophagopathy could no longer be ruled out, even in the absence of symptoms [4].

Thus, it is essential to emphasize the need for development for structural actions aimed at increasing rates of early diagnosis followed by etiological treatment in the acute form of the disease, reducing the significant number of diagnoses in the chronic phase, in which time quality of life and life expectancy have already been compromised. From that, lies the importance of the Chagas Disease Clinic of the State University of Rio Grande do Norte as a specialized service, part of the Brazilian public health system, dedicated to care for these patients and promote a cost‐free diagnosis, treatment, and follow‐up. Also, the service provides adequate health support in an endemic region of the country with people that have a vulnerable socioeconomic condition and few resources to access a private health facility.

Diagnosis was confirmed by an esophagogram, which provided evaluation of the morphofunctional appearance of the organ and enabled staging the disease using Rezende’s classification. The prevalence profile of different stages of megaesophagus in the region where the patient is located for Stages I, II, III, and IV of Rezende is 53.8%, 30.8%, 7.7%, and 7.7%, respectively [4]. Being classified as Rezende’s Group III, the patient was sorted into one of the less common groups at diagnosis.

The treatment focuses on controlling symptoms and improving the patient’s swallowing function. It includes medications to reduce lower esophageal sphincter pressure, eating habits changes, and interventional procedures, which become more invasive as the disease advances [13, 14]. Also, isolated clinical treatment is only recommended for oligosymptomatic patients, those with high surgical risk, surgery refusal, or when surgery is unavailable due to its reduced efficacy compared to interventional treatment [13].

Finally, laparoscopic cardiomyotomy surgery was the therapy of choice due to its wide application and proven efficacy in cases of nonadvanced megaesophagus (Rezende’s Groups I, II, and III), showing great outcomes in 90% of these patients [15, 16]. After surgery she no longer reported any esophageal symptoms, has been eating without restrictions and is keeping a stable weight according to data from her last doctor’s appointment.

## 4. Conclusion

The challenges related to Chagas disease are numerous and multifactorial, including clinical and epidemiological aspects. As a neglected disease, it results in high rates of underreporting and a socioeconomic impact in which the main groups at‐risk are those who have the least access to healthcare services, further hindering early diagnosis and quality follow‐up.

Both clinical presentation and exam results of the patient were consistent with what is described in the literature. Additionally, the radiographic findings showed good compatibility with Rezende’s radiographic staging, which contributed to choosing the most appropriate surgical treatment.

Additionally, the effectiveness of laparoscopic Heller myotomy over symptom control can also be highlighted once it restores the patient’s esophageal function, improving her health and quality of life.

Finally, although this disease was discovered over a century ago, it still holds questions that require further research and scientific explanation, whether to better comprehend the disease’s onset or also the lack of correlation between symptoms and esophageal dilation. This study sought to discuss these points, drawing on the case report of a patient who manifested a late Chagas esophagopathy.

## Funding

The author José Antonio da Silva Júnior was a master’s fellow supported by the Coordination for the Improvement of Higher Education Personnel (number process: 88887.671123/2022‐00) and a PhD fellow funded by the National Council for Scientific and Technological Development (number process: 141276/2024‐0).

## Ethics Statement

This study was approved by the State University of Rio Grande do Norte Research Ethics Committee under approval no. 6.527.976/2023 (CAAE: 74386523.8.0000.5294) and was conducted with patients’ authorization by means of a signed consent form.

## Conflicts of Interest

The authors declare no conflicts of interest.

## Data Availability

The data that support the findings of this study are available on request from the corresponding author. The data are not publicly available due to privacy or ethical restrictions.
